# Subtle but Significant: Intraspecific Genome Size Variation in Durum Wheat Landraces and Cultivars

**DOI:** 10.3390/plants15172717

**Published:** 2026-09-04

**Authors:** Ahmet Gökçebel, Angelos C. Kyratzis, Nikolaos Nikoloudakis

**Affiliations:** 1 Department of Agricultural Science, Biotechnology and Food Science, Cyprus University of Technology, Limassol 3036, Cyprus; ai.gokcebel@edu.cut.ac.cy; 2 Agricultural Research Institute, Ministry of Agriculture, Rural Development and Environment, Nicosia 1516, Cyprus; akyratzis@ari.moa.gov.cy

**Keywords:** Cypriot landraces, flow cytometry, genetic diversity, *Triticum turgidum* ssp. *durum*

## Abstract

Genome size is a significant trait connected to the evolutionary history of plants and frequently linked to speciation events. In the current study, we evaluated intraspecific variation in 2C-values across a durum wheat collection comprising landraces and cultivars of diverse origins, preserved in the Cypriot gene bank. Forty-seven accessions were analysed by Propidium Iodide (PI) staining/flow cytometry (FCM), and subtle differences were noted. 2C content varied from 25.04 pg for Kyperounta landrace to 26.78 pg for ARI00068. In general, 2C value overlap was observed across the collection, although some accessions were distinguished. Comparison among landraces and modern varieties indicated that landraces exhibit a broader range of 2C content than cultivars, but a clear clustering based on geographic origin was not observed. The genome size data provided here could offer an additional layer of information for germplasm collections and serve as a stepping stone for further omics analyses.

## 1. Introduction

*Triticum turgidum* ssp. *durum* (durum wheat) is essential to maintaining food security worldwide. It accounts for 8–10% of the total wheat-growing area and is grown on around 18 million hectares worldwide, yielding an estimated 35–40 million metric tons per year [1]. About 60% of the world’s durum wheat is produced in the Mediterranean Basin and Canada, where it is mostly used for pasta, couscous, bulgur, and some types of bread [2]. Durum wheat is a crucial crop for regions currently experiencing climate stress since it is more suited to semi-arid and hot climates than *T. aestivum* (bread wheat) [3]. Wheat yield sufficiency is currently threatened by rising demand due to population increase, climate change, and environmental degradation; thus, underlining the need for more resilient/high-yielding cultivars under unfavorable conditions [4].

Beginning with the cultivation of *T. turgidum* ssp. *dicoccoides* (wild emmer), durum wheat was domesticated in the Fertile Crescent approximately 12,000 years ago. This was followed by a slow shift towards non-brittle spikes and free-threshing grains [5,6,7,8,9]. While modern breeding has led to significant improvements in yield and quality, it has also caused a narrowing of the genetic base due to repeated selection for a limited number of traits, often at the expense of diversity [10]. This reduction in genetic variation has limited breeders’ ability to respond to emerging environmental and biological challenges. Although historical landraces possessed rich genetic diversity and resilience to stress, their lower yield, lodging susceptibility, and poorer technological traits in some cases resulted in their replacement by high-performing modern cultivars [11,12].

Genome size, commonly expressed as the C-value/2C-value, represents the amount of DNA contained within a haploid set of chromosomes and is a fundamental attribute of eukaryotic organisms. In practice, nuclear DNA content is often reported as 2C, which corresponds to the DNA amount in a non-replicated somatic nucleus (G1 phase) and serves as a standard reference in flow cytometry-based genome size estimation. Importantly, the terminology describing 2C-values is independent of ploidy level, meaning that these are routinely used even in polyploid species [13]. In plants, genome content varies extensively across species, yet this variation shows no consistent relationship with organismal complexity. This discrepancy, referred to as the “C-value paradox” or “C-value enigma”, reflects the fact that genome size differences are largely driven by variation in repetitive and non-coding DNA rather than differences in gene(s) number [14,15,16]. Although not directly related to organismal complexity, genome size plays a significant role in shaping structural and functional attributes. At the cellular level, larger genomes are typically linked to increased nuclear and cell size, as well as prolonged cell cycle duration, reflecting nucleotypic effects of DNA content. These effects can influence higher-level biological processes, including growth rate, developmental timing, and life-history strategies [17]. For instance, species with smaller genomes often exhibit faster growth and shorter generation times, whereas larger genomes are generally associated with slower development [18]. Genome size variation also appears to play a role in ecological adaptation and physiological performance, affecting characteristics such as biomass, seed features, and resilience to environmental stress [19,20]. Therefore, the C-value, whether expressed as 1C or measured as 2C in experimental contexts, represents not only a measure of DNA content but also an important factor shaping plant development, adaptation, and evolutionary trajectories.

Genome size variation represents an additional layer of biological diversity, observed across different levels of biological organization, and is commonly categorized as interspecific (between species) and intraspecific (within species) variation [21]. In plants, interspecific differences can be striking even among species with the same ploidy level, with reported nuclear DNA contents spanning several orders of magnitude (e.g., 0.14 to 304.46 pg/2C, as reported in Kew Plant DNA C-values Database; https://cvalues.science.kew.org/, assessed on 30 May 2026), and this broad divergence is largely attributed to the differential accumulation and deletion of repetitive DNA, particularly retrotransposons, whose proliferation can drive substantial genome expansion over evolutionary time. These genome size differences also have practical value, for example, as diagnostic markers in morphologically similar groups such as wild wheats, where flow cytometry can distinguish species based on 2C-values [22,23]. Intraspecific genome size variation can occur among individuals, populations, or even between sub-genomes in polyploids, arising through mechanisms such as chromosomal changes, structural variants, introgression, and polymorphisms in repetitive elements. Although typically smaller in magnitude (often ~1–2% or less in many species) and still debated in extent, intraspecific variation is increasingly relevant for interpreting genome evolution [24].

The wheat genome is characterized by its large size and intricate evolutionary history, shaped by repeated polyploidization events and the extensive presence of repetitive DNA, which accounts for roughly 80% of its sequence [25]. Within the genus *Triticum*, tetraploid species such as *Triticum turgidum* ssp. *dicoccoides* and *Triticum timopheevii* ssp. *araraticum* display notable differences in genome size. For example, the DNA content (2C value) of *T. dicoccoides* typically falls between 25.206 and 25.834 pg, whereas *T. araraticum* exhibits smaller values ranging from 23.226 to 23.458 pg [26]. This corresponds to an interspecific difference of nearly 2 pg/2C (approximately 9.7%), largely explained by unequal accumulation of retrotransposable elements following their evolutionary divergence [26]. Similarly, the ‘Svevo’ genome of durum wheat, comprising the A and B subgenomes, is ~20.90 Gb (2C) in size and contains >85% repetitive content [27]. Although recent advances in sequencing technologies have enabled the generation of high-quality genome assemblies, important gaps remain. Inconsistencies between genome-size estimation methods and the limited understanding of recombination dynamics in repeat-dense pericentromeric regions continue to pose challenges [26,27]. Addressing these issues is essential for effectively leveraging the genetic diversity of wild wheat relatives in breeding programs aimed at enhancing crop performance and stress resilience.

Flow cytometry is a high-throughput analytical technique that measures optical signals from individual particles (e.g., isolated plant nuclei) as they are transported in a fluid stream past a laser, enabling rapid quantification of DNA-associated fluorescence (and scatter) on a particle-by-particle basis [28]. In plant genome-size applications, sample preparation commonly involves mechanical homogenization of plant tissue in a nuclei-isolation buffer, filtration to obtain intact nuclei, stoichiometric staining of nuclear DNA with fluorochromes such as propidium iodide, and estimation of genome size by comparing the sample’s fluorescence peak(s) to an internal reference standard [29]. Method performance in agricultural species is strongly influenced by buffer chemistry, fluorochrome choice, tissue type, reference standards, and plant secondary metabolites (e.g., phenolics/tannins) that can interfere with staining and fluorescence, increasing uncertainty in absolute genome-size estimates if protocols are not controlled [28,29]. Building on the general use of flow cytometry for plant DNA analysis, wheat provides clear examples of how the method supports both breeding-oriented ploidy screening and genome-level chromosome analysis.

In bread wheat, laser flow cytometry has been applied to rapidly determine the ploidy of plants regenerated from microspore/anther culture by measuring nuclear DNA content in PI-stained leaf nuclei, using internal standards and collecting fluorescence signals from nuclei in the G0/G1 phase; this approach distinguished haploid (n = 3x), doubled-haploid/hexaploid (2n = 6x), and higher endopolyploid classes [30]. In durum wheat, flow cytometry has also been extended to flow cytogenetics, where DAPI (4′,6-diamidino-2-phenylindole) stained metaphase chromosomes are analyzed to generate “flow karyotypes” and then physically sorted, demonstrating that large-insert DNA libraries can be built from flow-sorted chromosomal DNA [31].

Despite the agronomic importance and genetic diversity of durum wheat landraces, limited information is available regarding genome size variation among durum/turgidum wheat germplasm [32,33,34], with even less information regarding intra-varietal diversity. Due to its geographic proximity to the Fertile Crescent, Cyprus is an important region for the conservation of durum wheat landraces shaped by millennia of cultivation under Mediterranean conditions. The unique geography and being at the crossroads of Near East and Mediterranean agriculture resulted in the uniqueness of Cypriot germplasm [29,35,36,37,38,39]. Moreover, there is a clear shift towards sustainability, and nowadays the European Commission and the CAP (2027) aim to valorize and conserve these diverse genetic resources.

Building on the current understanding of genome size variation, this study aimed to examine 2C nuclear DNA content across a diverse set of durum wheat germplasm conserved in the national genebank. The primary objective was to estimate the 2C-values of modern cultivars and landraces using flow cytometry and to assess the extent of intraspecific variation within cultivated durum wheat. By comparing these groups, we further evaluated whether the distribution and variability of nuclear DNA content differed between landraces and modern cultivars. This characterization provides a baseline for documenting 2C-value diversity within durum wheat germplasm and for identifying accessions with contrasting nuclear DNA contents. Integration of these data with phenotypic, environmental, and genomic information in future studies could help determine the underlying basis and potential biological significance of the observed variation.

## 2. Materials and Methods

### 2.1. Plant Material

Plants were grown in pots with Plantaflor Blocking Compost (Münsterstr. 17, 49377 Vechta, Germany) under a long-day (16 h light/8 h dark) photoperiod at 19–22 °C. When plants reached the tillering stage, leaf samples were collected. Freshly cut leaves were then placed between wet paper towels and stored in zipper bags at a low temperature (8 °C), and all analyses were conducted within 3 days using the same batch of buffer (Table 1). Plant seeds used as an internal flow standard (*Pisum sativum* cv. Ctirad; 2C = 9.09) were obtained from Prof Doležel/the Center of Plant Structural and Functional Genomics (Šlechtitel ˚u 31, Holice, 77900 Olomouc, Czechia) and selected based on genomic size proximity to *Triticum turgidum* ssp. *durum* reported values. The study comprised 47 durum wheat accessions, including 38 landraces and 9 modern varieties (Table 2). The Cypriot accessions were obtained from the Cypriot genebank (accession codes as ARI; passport data in the Eurisco database; https://eurisco.ipk-gatersleben.de/, assessed on 30 May 2026), where they are maintained as part of the national durum wheat germplasm collection. Additional Mediterranean ICARDA-derived material included accessions originating from Greece, Italy, Spain, Algeria, Armenia, Morocco, Libya, Syria, Israel, and Jordan (accessions coded as Icarda; passport data in Genesys database; https://www.genesys-pgr.org/, assessed on 30 May 2026). All other varieties (released cultivars) are maintained in the ARI institute. Status (landrace or modern variety), geographical origin, and available accession identifiers are reported in Table 2.

### 2.2. Sample Preparation

In a Petri dish (placed on top of ice), a leaf area of approximately 0.5 cm^2^ per wheat sample and standard was chopped together for approximately 10 s using a sterile double-edged razor blade per sample. The tissues were immersed in one milliliter of pre-chilled buffer (Table 1). Homogenates were passed through 30 µm Celltrics nylon filters (Sysmex, Lincolnshire, IL, USA) into 1.5 mL Eppendorf tubes and held at 8 °C for 10 min to increase/improve staining. A total of 47 wheat accessions were analyzed as biological replicates (three independent plants per accession).

**Table 1:** Composition of nuclei isolation buffers used for wheat analyses [29].

Buffer	Chemical Composition
Sorbitol-Based Buffer (SBB)	100 mM Tris-HCl; 0.35 M Sorbitol; 0.05 M glycine; 5 mM EDTA; 90 mM NaCl; 1% (*w*/*v*) Polyvinylpyrrolidone (PVP-40); 0.5% (*v*/*v*) Tween 20; 0.1% (*v*/*v*) β-mercaptoethanol; 50 µg/mL RNAse; 50 µg/mL Propidium Iodide; pH 7.5

### 2.3. Flow Cytometry

The efficiency of the buffer in isolating nuclei and enabling accurate 2C-value estimation was evaluated using a BD Accuri C6 flow cytometer (Accuri Cytometers, Inc., Ann Arbor, MI, USA), following the methodology previously described [40]. Measurements were based on light-scattering and fluorescence signals generated by a 20 mW laser operating at 488 nm. Instrument performance and low CV histograms were verified using 8-peak Spherotech fluorescent calibration beads, according to the manufacturer’s guidelines (CFlow User Guide, Accuri). To minimize interference from debris, thresholds were set at 80,000 for FSC-H and 1000 for FL-2. Samples were analyzed at a low flow rate, with data acquisition limited to 2 min, corresponding to approximately 2000–4000 nuclei per run. Nuclei were selectively gated along a diagonal axis using SSC versus FL2-A and FL3-A versus FL2-A plots, and fluorescence peaks were visualized using count versus FL2-A histograms. Each accession was analyzed using three independent biological replicates (three individual plants). All measurements were completed within three consecutive days using the same freshly prepared buffer batch to ensure buffer freshness and minimize methodological variation. The resulting samples showed high consistency with minimal systematic variation. For 2C-value estimation, flow cytometry data were exported and analyzed using ModFit LT version 5.0 (Verity Software House, Topsham, ME, USA). The resulting histograms displayed well-defined peaks with coefficients of variation well below 3% on average, indicating high-quality measurements. Analyses were exported as PDF files, and R was used to extract FL2 values and CVs (Supplementary Data).

The genomic content of each cultivar was determined using the following formula:

$$2C\mathrm{nuclear}\mathrm{DNA}\mathrm{content}\mathrm{of}\mathrm{sample}\;(pg)\;=\frac{sample\;G0/G1\;mean\;FL\times 2C\;nuclear\;DNA\;content\;of\;reference\;standard}{\mathrm{reference}\mathrm{standard}G0/G1\mathrm{mean}FL}$$

### 2.4. Statistical Analyses

2C-value means and standard deviations (SDs) were calculated for each accession across the three independent biological replicates and are reported in Table 2. Replicate-level 2C-value measurements and associated raw flow-cytometry data are provided in the Supplementary Data. A one-way ANOVA was used to analyze differences among accessions, followed by Tukey’s honest significant difference (HSD) test. The *t*-test and Levene’s test were performed using the accession-level mean 2C nuclear DNA content, with each accession represented by the mean of its three biological replicates. Compared groups referred to accessions characterized as cultivars vs. landraces. The car, dplyr, multcompView, tidyr, and purr libraries and Rstudio (version 1.1.463) were used for the analyses (Supplementary Data), and SRplot [41] for the visualization of the heat map and *t*-test.

**Table 2:** List of wheat accessions used in this study, their geographical origin, and relevant cytogenetic and flow-cytometric traits. Values are presented as mean ± standard deviation. Statistical differences among varieties were assessed using Tukey’s honestly significant difference (HSD) test following analysis of variance (ANOVA). Different letters indicate statistically significant differences between means at *p* < 0.05; values sharing the same letter are not significantly different. The table reports measured 2C nuclear DNA content (pg), estimated 2C nuclear DNA-content equivalents (Mbp), and coefficients of variation (CV) for wheat and the internal standard. One picogram (pg) of DNA corresponds to 978 megabase pairs (Mbp), following Doležel et al. [42].

No	Accession	Origin	2C (pg)	Pisum CV%	Triticum CV%	Mbp
1	ARI00007	Cyprus	25.99 ± 0.09 ^c–h^	2.48 ± 0.17	2.59 ± 0.25	25,416.86 ± 90.55 ^c–h^
2	ARI00009	Cyprus	25.97 ± 0.06 ^c–i^	2.89 ± 0.67	2.78 ± 0.65	25,398.12 ± 54.68 ^c–i^
3	ARI00015	Cyprus	25.96 ± 0.09 ^d–i^	2.35 ± 0.13	2.34 ± 0.05	25,385.49 ± 85.97 ^d–i^
4	ARI00017	Cyprus	25.76 ± 0.02 ^g–l^	2.01 ± 0.32	2.31 ± 0.12	25,193.95 ± 19.54 ^g–l^
5	ARI00027	Cyprus	25.87 ± 0.03 ^e–k^	2.54 ± 0.88	2.50 ± 0.68	25,300.45 ± 31.53 ^e–k^
6	ARI00031	Cyprus	26.22 ± 0.05 ^b–d^	2.44 ± 0.22	2.13 ± 0.14	25,642.55 ± 51.54 ^b–d^
7	ARI00048	Cyprus	26.26 ± 0.04 ^bc^	1.93 ± 0.08	2.02 ± 0.15	25,686.99 ± 41.75 ^bc^
8	ARI00052	Cyprus	25.91 ± 0.07 ^e–j^	2.00 ± 0.17	2.30 ± 0.24	25,336.88 ± 70.17 ^e–j^
9	ARI00053	Cyprus	25.70 ± 0.24 ^h–l^	2.64 ± 1.15	2.26 ± 0.17	25,131.20 ± 233.66 ^h–l^
10	ARI00058	Cyprus	25.96 ± 0.09 ^d–i^	3.10 ± 0.98	2.89 ± 0.82	25,389.39 ± 92.24 ^d–i^
11	ARI00067	Cyprus	26.31 ± 0.06 ^b^	2.60 ± 0.14	2.39 ± 0.06	25,734.98 ± 63.54 ^b^
12	ARI00068	Cyprus	26.78 ± 0.03 ^a^	3.49 ± 0.34	2.84 ± 0.47	26,190.06 ± 25.41 ^a^
13	ARI00070	Cyprus	26.17 ± 0.07 ^b–e^	2.22 ± 0.14	2.27 ± 0.31	25,591.57 ± 72.42 ^b–e^
14	ARI00075	Cyprus	25.92 ± 0.04 ^e–j^	2.45 ± 0.55	2.29 ± 0.83	25,345.75 ± 36.49 ^e–j^
15	ARI00077	Cyprus	26.22 ± 0.08 ^b–d^	3.35 ± 0.73	3.53 ± 0.33	25,642.18 ± 77.33 ^b–d^
16	ARI00079	Cyprus	25.93 ± 0.02 ^d–j^	1.99 ± 0.21	2.24 ± 0.16	25,354.82 ± 21.97 ^d–j^
17	ARI00086	Cyprus	25.97 ± 0.06 ^c–i^	1.91 ± 0.10	2.31 ± 0.09	25,403.18 ± 56.27 ^c–i^
18	ARI00090	Cyprus	25.73 ± 0.08 ^g–l^	3.28 ± 0.44	2.96 ± 0.64	25,166.09 ± 77.63 ^g–l^
19	ARI00093	Cyprus	25.86 ± 0.08 ^f–k^	2.66 ± 0.22	2.57 ± 0.11	25,295.40 ± 73.43 ^f–k^
20	ARI00100	Cyprus	25.78 ± 0.09 ^f–l^	2.31 ± 0.20	2.11 ± 0.12	25,216.16 ± 91.60 ^f–l^
21	ARI00101	Cyprus	25.84 ± 0.07 ^f–k^	2.08 ± 0.27	2.31 ± 0.05	25,273.90 ± 66.95 ^f–k^
22	ARI00102	Cyprus	26.08 ± 0.03 ^b–f^	2.08 ± 0.14	1.95 ± 0.04	25,508.00 ± 30.08 ^b–f^
23	ARI00105	Cyprus	26.00 ± 0.08 ^c–g^	1.93 ± 0.26	2.29 ± 0.26	25,430.27 ± 75.20 ^c–g^
24	Atlas ^1^	Greece	25.76 ± 0.04^g–l^	2.54 ± 0.18	2.51 ± 0.06	25,195.32 ± 34.65 ^g–l^
25	Famira	Cyprus	25.73 ± 0.09 ^g–l^	3.26 ± 0.64	3.83 ± 0.55	25,162.30 ± 85.71 ^g–l^
26	Hekabe ^1^	Cyprus	25.93 ± 0.02 ^d–j^	2.52 ± 0.15	2.43 ± 0.17	25,357.16 ± 20.13 ^d–j^
27	Icarda 1 (IG 97359)	Algeria	25.75 ± 0.09 ^g–l^	2.65 ± 0.86	2.88 ± 0.80	25,187.69 ± 89.54 ^g–l^
28	Icarda 2 (IG 126364)	Armenia	25.65 ± 0.03 ^j–l^	2.68 ± 0.17	2.57 ± 0.07	25,085.39 ± 25.31 ^j–l^
29	Icarda 3 (IG 96437)	Morocco	25.74 ± 0.02 ^g–l^	2.44 ± 0.03	1.95 ± 0.08	25,175.06 ± 21.41 ^g–l^
30	Icarda 4 (IG 98726)	Libya	25.68 ± 0.03 ^i–l^	2.39 ± 0.05	2.25 ± 0.05	25,111.46 ± 32.83 ^i–l^
31	Icarda 5 (IG 95789)	Syria	25.79 ± 0.06 ^f–l^	2.76 ± 0.76	2.38 ± 0.79	25,226.87 ± 63.17 ^f–l^
32	Icarda 6 (IG 84979)	Spain	25.81 ± 0.06 ^f–k^	2.29 ± 0.05	2.18 ± 0.26	25,239.64 ± 56.12 ^f–k^
33	Icarda 7 (IG 86653)	Israel	25.89 ± 0.05 ^e–k^	2.31 ± 0.09	2.09 ± 0.03	25,315.85 ± 46.35 ^e–k^
34	Icarda 8 (IG 97193)	Jordan	25.88 ± 0.09 ^e–k^	2.47 ± 0.18	2.76 ± 0.47	25,313.55 ± 92.81 ^e–k^
35	Icarda A(IG 85710)	Greece	25.78 ± 0.09 ^g–l^	2.93 ± 0.60	2.88 ± 0.50	25,212.81 ± 83.69 ^g–l^
36	Iride ^1^	Italy	25.65 ± 0.11 ^j–l^	3.85 ± 0.43	3.98 ± 0.46	25,086.83 ± 104.07 ^j–l^
37	Josephina ^1^	Cyprus	25.63 ± 0.11 ^j–m^	3.24 ± 0.76	3.08 ± 0.79	25,065.43 ± 104.22 ^j–m^
38	Kholina ^1^	Cyprus	25.63 ± 0.02 ^j–m^	2.82 ± 0.45	3.09 ± 0.07	25,065.45 ± 15.72 ^j–m^
39	Kyperounta	Cyprus	25.04 ± 0.01 ^o^	3.16 ± 0.18	2.92 ± 0.01	24,491.47 ± 5.83 ^o^
40	MHA	Greece	25.50 ± 0.12 ^l–n^	3.38 ± 0.08	4.24 ± 0.06	24,934.45 ± 116.88 ^l–n^
41	MHM	Greece	25.33 ± 0.20 ^m–o^	3.58 ± 0.29	3.88 ± 0.39	24,773.46 ± 193.17 ^m–o^
42	Macedonia ^1^	Cyprus	25.82 ± 0.17 ^f–k^	3.16 ± 0.61	3.04 ± 0.29	25,252.19 ± 166.21 ^f–k^
43	Mazotos ^1^	Cyprus	25.65 ± 0.07 ^j–l^	2.59 ± 0.27	2.80 ± 0.01	25,088.94 ± 64.98 ^j–l^
44	Ourania ^1^	Cyprus	25.87 ± 0.27 ^e–k^	3.48 ± 1.66	3.28 ± 0.66	25,305.03 ± 261.41 ^e–k^
45	Tripolitis Red	Cyprus	25.34 ± 0.03 ^m–o^	2.93 ± 0.11	2.81 ± 0.15	24,779.64 ± 28.21 ^m–o^
46	Tripolitis White	Cyprus	25.20 ± 0.03 ^no^	3.16 ± 0.04	3.19 ± 0.19	24,642.26 ± 26.87 ^no^
47	Vitron ^1^	Spain	25.60 ± 0.04 ^k–m^	2.49 ± 0.21	2.56 ± 0.05	25,040.53 ± 38.49 ^k–m^
	Average		25.82 ± 0.28	2.68 ± 0.50	2.67 ± 0.57	25,258.27 ± 282.40

^1^ Indicates modern variety.

## 3. Results

**Figure 1:**
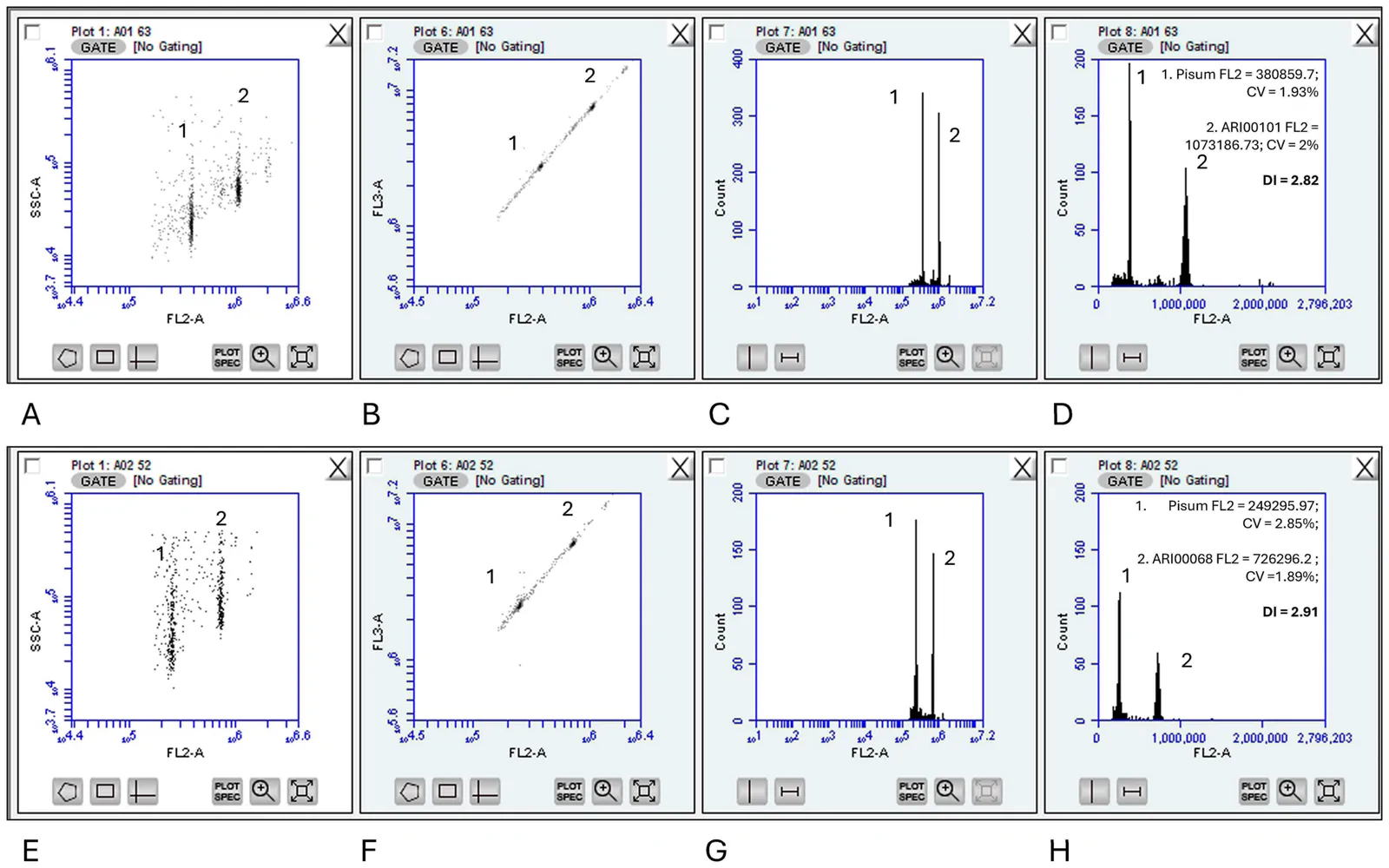
Representative gated flow cytometry (FCM) histograms and paired visualizations. Panels (**A**–**D**) display data for ARI00101, while panels (**E**–**H**) present the corresponding plots for ARI00068, with each letter pair (**A** and **E**, **B** and **F**, **C** and **G**, **D** and **H**) showing the same plot type for the two varieties. Numbered peaks within the histograms indicate modal populations, where 1 = *Pisum sativum* cv Ctirad and 2 = *Triticum turgidum* ssp. *durum* wheat. Panel (**A**,**E**) depict SSC-A vs. FL2-A gated scatter plots, panel (**B**,**F**) show FL3-A vs. FL2-A gated scatter plots, (**C**,**G**) display Count vs. FL2-A histograms plotted on a logarithmic FL2 axis, while panel (**D**,**H**) show Count vs. FL2-A histograms on a linear FL2 axis.

2C-values estimated for different accessions were adjusted to correspond with the genome size of the appropriate reference internal standard (Figure 1; Table 2). In every instance, the 2C peak (FL2-A axis) of each accession was observed next to the 2C peak of the reference (*Pisum sativum* cv. Ctirad), aiding in accurate DNA content assessments. That pattern was evident in both scatter-based gating (SSC-A vs. FL2-A; FL3-A vs. FL2-A) and in the fluorescence histograms, which allowed seamless gating. In all acquisitions, histograms/peaks were clear, and a low number of debris was detectable; thus confirming the consistency of sample(s) preparation, as well as optimal nuclei isolation capacity. Flow cytometry analysis revealed a relatively narrow range of nuclear DNA content among the evaluated accessions, with 2C values ranging from 25.04 pg in the Cypriot landrace Kyperounta to 26.78 pg in accession ARI00068 (Table 2). Nonetheless, in some cases, derived fluorescence ratios (DI values) differed significantly among accessions (Figure 1). The average 2C DNA content across all accessions was approximately 25.82 pg, corresponding to an average genome size of about 25,258 Mbp.

The quality and consistency of the flow cytometric measurements were supported by the relatively low coefficients of variation (CVs) obtained for both internal standard and durum wheat accessions. *Pisum sativum* CV values ranged from 1.91% (ARI00086) to 3.85% (Iride), while *Triticum turgidum* ssp. *durum* CV values ranged from 1.95% (ARI00102 and Icarda 3) to 4.24% (MHA). Overall, most accessions exhibited CV values below 3%, indicating high-quality histograms and reliable estimations of nuclear DNA content (Table 2). Accessions such as ARI00048, ARI00102, ARI00017, and Icarda 3 showed particularly low CVs, reflecting excellent sample preparation and measurement precision. In contrast, slightly elevated CV values observed in accessions including MHA, MHM, Famira, and Iride could indicate increased sample heterogeneity or minor variation during nuclei isolation and staining.

Cypriot and foreign accessions clustered around the mean value (25.82), indicating a generally conserved genome size within the studied germplasm. Overall, the ANOVA grouping indicated that most accessions fell into overlapping classes for 2C DNA content and genome size, suggesting that differences among most accessions were not statistically significant. Most accessions shared multiple grouping letters (e.g., c–k, g–l, b–g), reflecting a high degree of similarity in nuclear DNA content. Although considerable overlap was observed among Tukey HSD groups, the analysis identified several accessions with significantly contrasting 2C-values, particularly at the extremes of the distribution. Thus, 2C-value variation was sufficient to detect differences among selected accessions but provided limited discriminatory resolution among accessions with closely overlapping nuclear DNA contents; hence, cultivar differentiation is not feasible solely on 2C-value genomic content. As a result, in the current collection, several accessions could be distinguished based on unique or clearly separated ANOVA classes (One-way ANOVA revealed a highly significant effect of accession on 2C nuclear DNA content (F_46_,_94_ = 31.09, *p* = 2.05 × 10^−40^). In particular, ARI00068 exhibited the highest 2C value (26.78 ± 0.03 pg) and 2C nuclear DNA content (26,190.06 ± 25.41 Mbp), forming the unique class “a”, clearly separating it from all other accessions. Conversely, Kyperounta showed the lowest values (25.04 ± 0.01 pg; 24,491.47 ± 5.83 Mbp) and was assigned to the distinct class “o”, indicating significant differentiation from the remaining material. Additional distinguishable accessions included Tripolitis White (class “no”), MHM and Tripolitis Red (class “m–o”), MHA (class “l–n”), Vitron (class “k–m”), and Josephina and Kholina (class “j–m”). At the upper end of the distribution, ARI00067 belonged to class “b”, while ARI00048 was grouped as “bc”, also showing partial statistical separation from the bulk of accessions. As a result, despite genome size variation among the studied germplasm being generally limited, a small number of accessions displayed statistically distinct nuclear DNA contents and could therefore be differentiated into unique ANOVA grouping classes.

**Figure 2:**
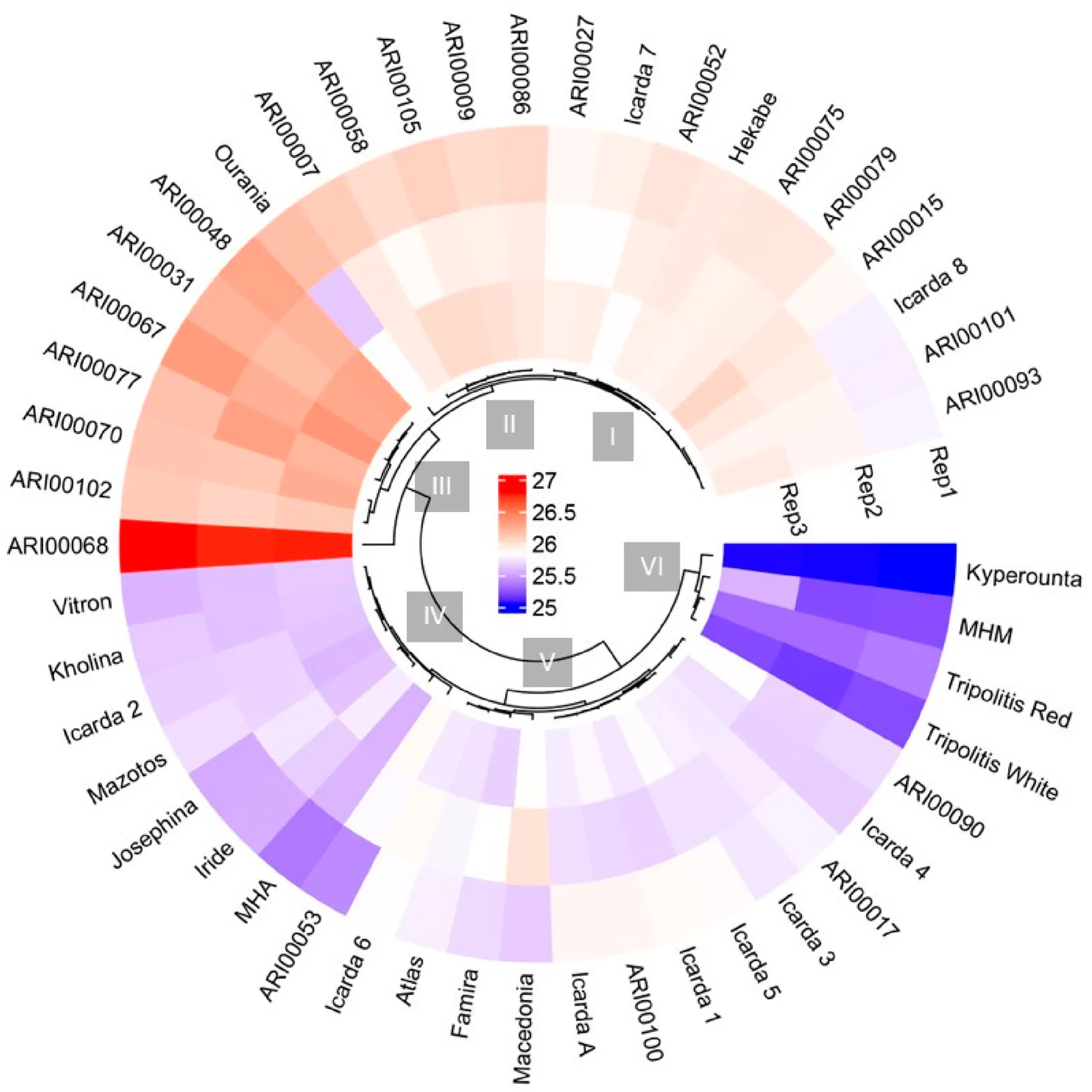
Circular heatmap depicting hierarchical clustering of wheat accessions based exclusively on nuclear DNA content (2C, pg). The outer ring lists individual accessions, while the inner one provides a descriptive grouping according to similarity in their measured 2C values. The heatmap employs a continuous colour gradient from blue (lower values, approximately 25 pg), through intermediate shades (25.5–26.5 pg), to red (higher values, approximately 27 pg). Accessions positioned on adjacent branches (I–VI) generally exhibit similar 2C-values, whereas greater branch separation and contrasting colours reflect larger differences in measured nuclear DNA content.

To further detect subtle affiliations, a composite heatmap hierarchical analysis was performed, yielding several distinct 2C-value clusters (Figure 2). Overall, genome size estimates across the collection revealed a narrow but structured range of 2C DNA content, and the circular heatmap provided a clear visualization of how quantitative variation aligns with hierarchical relationships that do not correspond to phylogenetic affiliations. The clustering pattern was dominated by a large central group composed primarily of Cypriot ARI accessions, most of which exhibited an intermediate range of 25.85–26.10 pg and tightly clustered heatmap colors. Within this cluster, accessions ARI00007, ARI00009, ARI00015, and ARI00052 showed highly similar 2C values and low CVs, indicating both 2C-value proximity and consistent flow cytometric performance. A distinct high-value subgroup emerged within the ARI collection, characterized by ARI00048, ARI00067, and especially ARI00068, which displayed the largest genome size in the dataset. These accessions formed a coherent red-shifted branch in the heatmap, suggesting a similar 2C-value range relative to the rest of the panel. In contrast, several Cypriot landraces clustered toward the lower end of the genome-size spectrum. Kyperounta, Tripolitis White, and Tripolitis Red consistently exhibited the smallest 2C values, forming a visually distinct blue-shifted cluster. Greek accessions MHA and MHM also fell within the lower-value region. Interestingly, despite no apparent grouping based on (landrace/cultivar) use or origin (Cypriot/foreign), the majority of cultivars were affiliated with one cluster (Kholina, Vitron, Mazotos, Josephina, Iride), having an intermediate 2C-value. Non-Cypriot ICARDA and other Mediterranean accessions were distributed across the heatmap rather than forming a single cohesive group, reflecting their diverse geographic origins. Several, including Icarda 1, Icarda 2, Icarda 4, and Icarda 5, aligned with the mid-range ARI cluster, whereas others such as Icarda 3 and Icarda 7 grouped with slightly lower-value Cypriot accessions.

**Figure 3:**
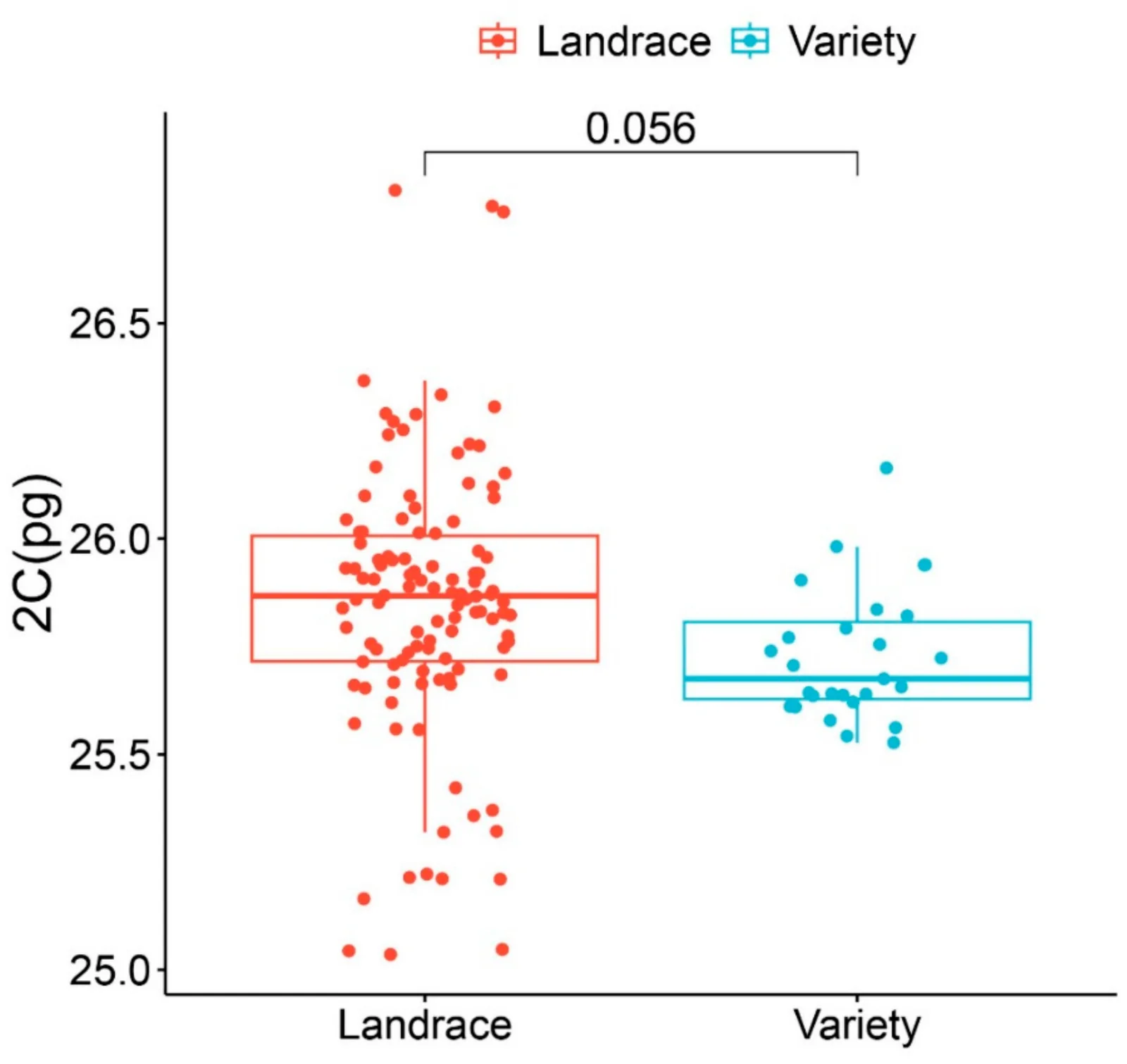
Comparison of nuclear DNA content between durum wheat landraces and modern varieties. Boxplots show the distribution of 2C values for accessions classified by use category, with individual data points overlaid to illustrate within-group variability. The bracket above the plot indicates the statistical comparison between groups, with a *p*-value of 0.056, suggesting a marginal but non-significant trend toward higher genome size in landraces.

To assess whether genome size variation is associated with the historical use category of durum wheat, 2C DNA content was compared between landraces and modern varieties. The boxplot analysis (Figure 3) showed that landraces tend to have slightly higher median 2C values than varieties, although the distributions remain broadly overlapping. Individual data points revealed that both groups span a similar range, indicating that neither category is characterized by extreme outliers or unusually constrained variability. The *t*-test statistical comparison yielded a *p*-value of 0.056, and an extended range of 2C-values in landraces as detected by Levene’s test for equality of variances (F = 5.4592; *p* = 0.021).

This pattern is consistent with the broader dataset, where several Cypriot landraces—such as Kyperounta, Tripolitis Red, and Tripolitis White—clustered at the lower end of the genome-size spectrum, while others aligned more closely with mid-range ARI accessions (Figure 2). In contrast, modern varieties showed a narrower central tendency and less spread in genome-size measurements than landraces.

## 4. Discussion

The present study investigated 2C-values variation among Cypriot and Mediterranean durum wheat germplasm using flow cytometry and revealed that, although overall variation in nuclear DNA content was relatively limited (Table 2), several accessions displayed statistically significant differentiation. The absolute nuclear DNA content was calculated using *Pisum sativum* cv. Ctirad as the internal reference standard, adopting the widely accepted calibration value (2C = 9.09 pg) established by Doležel et al. [13]. This calibration has served as the benchmark for plant flow-cytometric genome-size estimation for more than two decades and remains the basis for the majority of published genome-size studies, thereby ensuring comparability of our results with the existing literature and Kew C-value database. Recently, however, Soni and Henry [43] revisited the calibration of several commonly used flow cytometric reference standards using telomere-to-telomere (T2T) and haplotype-resolved genome assemblies. Their study suggests that the historical calibration of *P. sativum* cv. Ctirad (2C = 9.09 pg) may overestimate the absolute DNA content, proposing recalculated values of approximately 7.99 pg based on updated data.

Across all analyzed accessions, 2C values ranged from 25.04 pg to 26.78 pg, corresponding to an approximate genome size range of 24.5–26.2 Gbp. These findings are generally consistent with previously reported genome size estimates for tetraploid wheats [26,33,34] and further support the view that cultivated durum wheat exhibits a comparatively conserved genome structure despite its long evolutionary and breeding history [44]. Although the observed range of 2C-values was relatively narrow, the significant accession effect indicates measurable intraspecific variation in nuclear DNA content within the examined durum wheat germplasm. Nonetheless, the molecular basis of this variation cannot be determined from flow cytometry alone. In plant genomes, differences in nuclear DNA content may arise from variation in repetitive DNA, transposable elements, or other structural components of the genome; however, determining whether these mechanisms underlie the variation observed here would require complementary sequence-based or cytogenetic analyses. Similarly, the potential functional consequences of these 2C-value differences cannot be established without phenotypic validation. Thus, the present results should be considered a quantitative characterization of intraspecific 2C-value diversity and a basis for subsequent investigation of its molecular origin and potential biological significance.

The relatively narrow range of variation observed in the present study aligns with earlier reports, indicating that intraspecific genome size differences in crop species are usually modest compared with the much larger divergence observed among related species [19,45]. In *Triticum*, interspecific genome size differences are frequently associated with large-scale changes in repetitive DNA content, particularly the amplification and removal of retrotransposable elements following species divergence and polyploidization events [23,27,46]. For example, Özkan et al. [26] reported that differences between wild tetraploid wheats were strongly associated with differential retrotransposon accumulation, highlighting the dynamic nature of repetitive DNA during wheat genome evolution. The relatively narrow range observed within cultivated durum wheat is consistent with the possibility that domestication and modern breeding have contributed to the maintenance of relatively constrained variation in nuclear DNA content [10]. Polyploid genomes are also believed to possess buffering mechanisms that reduce the phenotypic consequences of structural genomic fluctuations, thereby allowing the maintenance of genomic integrity despite repetitive DNA turnover [47]. Nevertheless, the observed differences in 2C nuclear DNA content could potentially arise from variation in repetitive DNA composition, chromosomal rearrangements, copy-number variation, or introgression, although the present study does not allow these underlying mechanisms to be distinguished. Future genomic analyses could test whether any of these mechanisms contribute to the observed variation.

The biological significance of subtle genome size variation remains an important topic in plant evolutionary biology. Although genome size is not directly associated with organismal complexity, several studies have demonstrated that it can influence cellular and physiological characteristics through nucleotypic effects [18]. Previous studies have associated genome size with several nucleotypic characteristics, including nuclear and cell size, cell-cycle duration, developmental rate, and life-history traits [17,48]. Associations with biomass accumulation, seed characteristics, and responses to environmental conditions have also been reported across plant taxa [19,20]. These observations provide a broader biological context for investigating 2C-value variation within crop germplasm. However, developmental, physiological, and stress-response traits were not evaluated in the present study, and consequently no direct relationship between the 2C-value differences observed here and plant performance or stress adaptation can be inferred. Whether the detected intraspecific variation in nuclear DNA content is associated with phenotypic differences among durum wheat accessions therefore remains an open question requiring targeted phenotypic evaluation. In the context of durum wheat, which is cultivated extensively under semi-arid Mediterranean conditions, even relatively small differences in DNA content may influence developmental timing, biomass accumulation, or stress responsiveness. While the present study did not directly assess physiological traits, the observed variation among accessions might reflect specific genomic configurations. Similar hypotheses linking genome size variation with ecological adaptation have been proposed in several plant systems [20,21]. Future integration of genome size data with ecophysiological and agronomic traits could therefore help clarify whether such variation contributes functionally to stress adaptation in Mediterranean wheat germplasm.

Particularly noteworthy was the variation observed among several Cypriot accessions, including Kyperounta, Tripolitis White, Tripolitis Red, and ARI00068. Kyperounta exhibited the lowest 2C-value in the collection, whereas ARI00068 showed the highest value and formed an independent statistical group. Comparison of landraces and modern cultivars revealed no statistically significant difference in mean 2C nuclear DNA content (*p* = 0.056; Figure 3), whereas Levene’s test detected a significant difference in variance between the two groups (F = 5.46, *p* = 0.021), with landraces exhibiting greater dispersion in 2C-values. These results therefore indicate greater variability in nuclear DNA content among landraces rather than a difference in mean genome size. The historical cultivation of durum wheat in Cyprus and the island’s position between the Near East and the Mediterranean Basin provide relevant context for the germplasm examined; however, the present 2C-value data alone cannot determine whether the observed variation is associated with geographic origin, historical selection, or environmental adaptation.

It has been reported that in Mediterranean regions, durum wheat landraces originating from warm and arid environments typically display shorter growth cycles, reduced biomass, and lower grain weight compared to those from cooler and wetter regions, with climate variables explaining a substantial proportion of variation in traits such as days to anthesis (32.8%) and plant height (28.3%) [49]. High temperatures and evapotranspiration at the site of origin are further associated with increased tillering and spike number, but reduced grain filling and grain weight) [49]. Similarly, in Mediterranean bread wheat, agronomic performance follows a climatic gradient, with yield, grain-filling rate, and grain weight increasing from warm–dry southeastern regions toward cooler, wetter northern areas, while grain-filling duration decreases [50]. Genomic analyses support these patterns, as SNP-based studies using 13,177 markers have shown that population structure is largely influenced by temperature and radiation conditions around anthesis [50]. Comparable trends are observed in Chinese wheat landraces, where analyses across multiple agro-ecological zones using 52,303 markers revealed clustering patterns in which phenotypic groupings align closely with genotypic classifications, indicating strong co-adaptation to local environments [51].

In addition, previous studies have emphasized the uniqueness and high diversity of Cypriot wheat germplasm, likely resulting from centuries of farmer selection under heterogeneous Mediterranean environments [39]. Due to its environmental heterogeneity, relative geographic isolation, and long-standing agricultural traditions, Cypriot germplasm may have retained locally adapted traits that have been partially lost in more intensively bred modern cultivars. The clustering analysis further supported this interpretation by revealing structured groupings among accessions rather than random variation across the panel. Interestingly, several foreign ICARDA accessions clustered closely with Cypriot accessions. However, similarity or clustering based solely on 2C nuclear DNA content cannot be interpreted as evidence of shared ancestry, population structure, or convergent adaptation. Testing such hypotheses would require integration of the present 2C-value data with genome-wide marker or sequence data, environmental metadata, and standardized phenotypic information.

The comparison between landraces and modern cultivars indicated differences in the dispersion, rather than the mean, of nuclear DNA content. Although landraces exhibited a slightly higher median 2C value, the difference in mean 2C-value between the two groups was not statistically significant (*p* = 0.056). In contrast, the significant Levene’s test (F = 5.46, *p* = 0.021) demonstrated greater variance in 2C-values among landraces. Thus, the present results indicate that the landrace group encompasses a broader range of nuclear DNA content than the modern cultivars examined here. This greater 2C-value dispersion should not, however, be interpreted as evidence of greater genome-wide functional diversity, since sequence-level polymorphism, structural variation, and allelic diversity were not assessed. Although the statistical comparison did not meet the conventional significance threshold (*p* = 0.056), landraces tended to exhibit slightly broader variation and marginally higher median genome sizes than modern varieties. In contrast, modern breeding programs often involve repeated selection for high yield, uniformity, and technological quality, processes that can narrow the genomic base of cultivated material [10,11]. Such a reduction in diversity has been widely documented in wheat and other crop species and represents a major concern for long-term breeding resilience under climate change [4]. The greater dispersion of 2C-values observed among landraces indicates that they encompass a wider range of nuclear DNA contents than the modern cultivars examined here. This finding supports their value as material for continued germplasm characterization, although determining whether this broader 2C-value distribution corresponds to greater sequence-level genetic diversity, structural variation, or adaptive potential will require independent genomic and phenotypic analyses.

Another important outcome of this work is the demonstration of the effectiveness and reproducibility of flow cytometry for genome size estimation in wheat germplasm characterization. Most analyzed accessions exhibited coefficients of variation below 3%, indicating high-quality histograms and reliable fluorescence measurements. The successful application of the Sorbitol-Based Buffer and the use of *Pisum sativum* cv. Ctirad as an internal standard enabled consistent nuclei isolation and accurate DNA quantification across all samples. Given the large and highly repetitive nature of the wheat genome, obtaining stable and reproducible measurements can be technically challenging, particularly due to interference from secondary metabolites and staining variability [13,28,29]. The low CV values obtained in this study, therefore, confirm the robustness of the optimized protocol. Nonetheless, although the overall peak quality was high, accessions exhibiting comparatively higher CVs warrant greater caution when interpreting small differences in 2C-value. Higher peak CVs indicate reduced precision in determining the G0/G1 fluorescence peak position and may therefore affect the exact ranking of accessions with closely spaced 2C-values. Accordingly, the rank order of such accessions should not be overinterpreted, and greater emphasis should be placed on reproducibility across biological replicates and statistically supported differences between clearly contrasting accessions.

Similar studies in wheat have demonstrated the utility of flow cytometry not only for genome size estimation, but also for ploidy determination and chromosome sorting applications [52]. Beyond genome size estimation, flow cytometry represents a rapid and cost-effective tool for germplasm screening, ploidy determination, and cytogenetic analyses, with considerable potential for supporting breeding and conservation programs, at a fraction of the cost compared to omic technologies.

## 5. Conclusions

The present study demonstrates that genome size variation within durum wheat is relatively constrained, although measurable and statistically significant differences in 2C nuclear DNA content were detected among the examined germplasm in several accessions. These results provide a quantitative characterization of intraspecific C-value diversity within cultivated durum wheat and identify a limited number of accessions with comparatively distinct nuclear DNA-content estimates.

## Data Availability

The original contributions presented in this study are included in the article/Supplementary Material. Further inquiries can be directed to the corresponding author(s).

## Supplementary Materials

The following supporting information can be downloaded at: https://www.mdpi.com/article/10.3390/plants15172717/s1.
